# Supplementary material for: Splice-Junction-Based Mapping of Alternative Isoforms in the Human Proteome
Source: Cell Rep. Author manuscript; Available in PMC 2020 Jan 15. (PMC6961840; doi:10.1016/j.celrep.2019.11.026)
Supplement: 3 [file NIHMS1546469-supplement-3.zip › DF2/PXD0010154/Spleen-49-P24821-TPVLSAEASTAMGSPK.pdf]

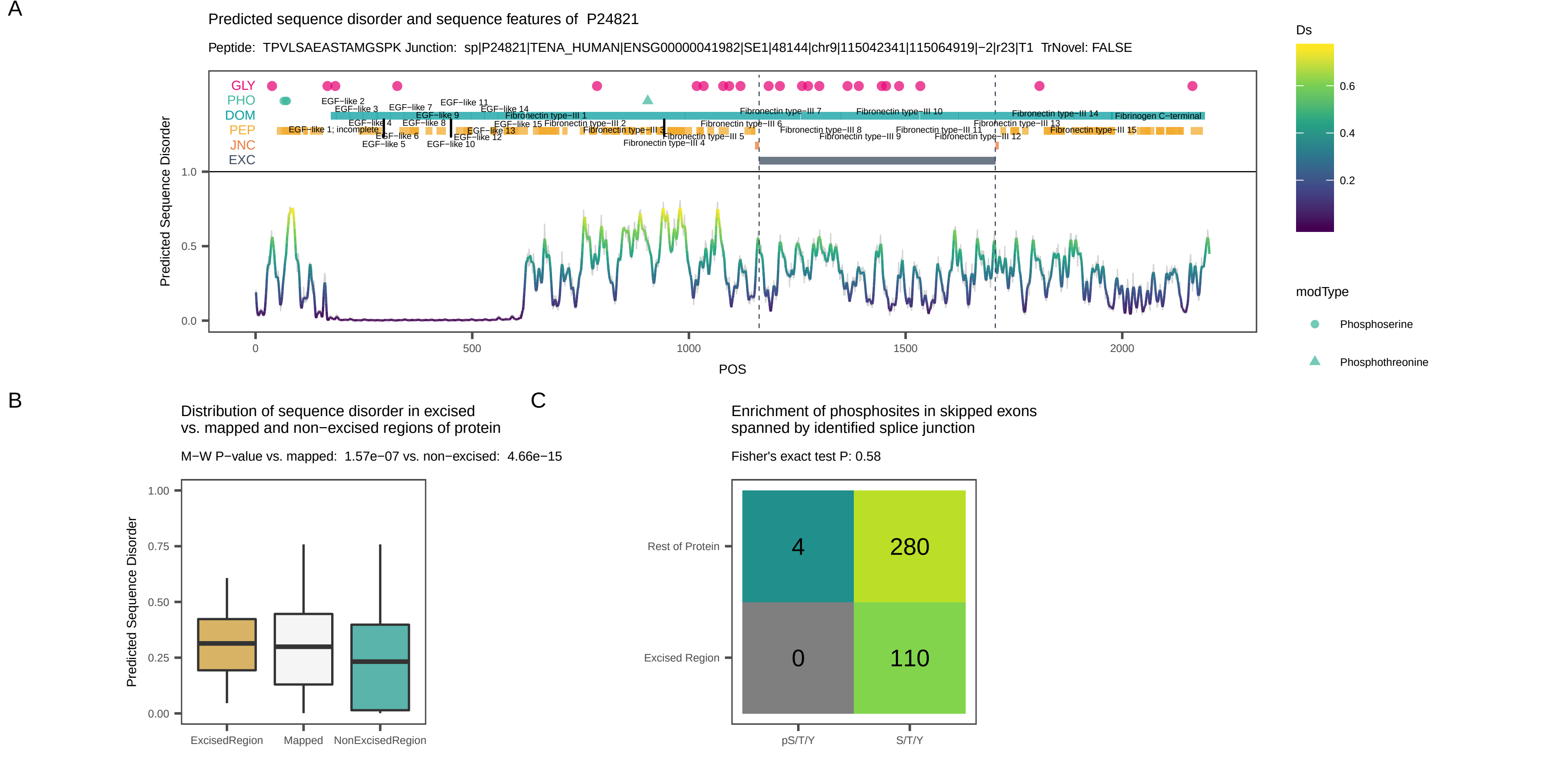
A
Predicted sequence disorder and sequence features of P24821
Peptide: TPVLSAEASTAMGSPK Junction: sp|P24821|TENA_HUMAN|ENSG00000041982|SE1|48144|chr9|115042341|115064919|−2|r23|T1 TrNovel: FALSE
GLY
PHO
DOM
PEP
JNC
EXC
EGF−like 1; incomplete
EGF−like 2
EGF−like 3
EGF−like 4
EGF−like 5
EGF−like 6
EGF−like 7
EGF−like 8
EGF−like 9
EGF−like 10
EGF−like 11
EGF−like 12
EGF−like 13
EGF−like 14
EGF−like 15
Fibronectin type−III 1
Fibronectin type−III 2
Fibronectin type−III 3
Fibronectin type−III 4
Fibronectin type−III 5
Fibronectin type−III 6
Fibronectin type−III 7
Fibronectin type−III 8
Fibronectin type−III 9
Fibronectin type−III 10
Fibronectin type−III 11
Fibronectin type−III 12
Fibronectin type−III 13
Fibronectin type−III 14
Fibronectin type−III 15
Fibrinogen C−terminal
Predicted Sequence Disorder
1.0
0.5
0.0
0
500
1000
1500
2000
POS
Ds
0.6
0.4
0.2
modType
Phosphoserine
Phosphothreonine
B
Distribution of sequence disorder in excised vs. mapped and non−excised regions of protein
M−W P−value vs. mapped: 1.57e−07 vs. non−excised: 4.66e−15
Predicted Sequence Disorder
1.00
0.75
0.50
0.25
0.00
ExcisedRegion
Mapped
NonExcisedRegion
C
Enrichment of phosphosites in skipped exons spanned by identified splice junction
Fisher's exact test P: 0.58
Rest of Protein
Excised Region
4
280
0
110
pS/T/Y
S/T/Y
